# 7.2 ELECTRORETINOGRAPHIC ANOMALIES SEEN IN PATIENTS AFFECTED BY SCHIZOPHRENIA OR BIPOLAR DISORDER ARE DETECTABLE EARLY IN CHILDREN BORN TO AN AFFECTED PARENT: IMPLICATIONS FOR THE STAGING OF RISK STATUS IN CHILDHOOD-ADOLESCENCE

**DOI:** 10.1093/schbul/sby014.024

**Published:** 2018-04-01

**Authors:** Michel Maziade, Thomas Paccalet, Anne-Marie Gagné, Elsa Gilbert, Daphné Lussier, Marc Hébert, Valérie Jomphe, Nathalie Gingras

**Affiliations:** 1 Centre de recherche CIUSSS de la Capitale-Nationale; 2 Université de Québec à Trois-Rivières et Centre de recherche CIUSSS-CN; 3 Université Laval & Centre de recherche CIUSSS-CN

## Abstract

**Background:**

Adult patients having schizophrenia, bipolar disorder or major depression display indicators of brain dysfunctions that may be detectable in healthy children-adolescents at genetic risk, such as those born to an affected parent (Maziade, New Eng J Med 2017; Schizophr Res 2013). For instance, cognitive deficits are displayed by both adult patients and children at risk (Maziade, Schizophr Bull 2011). We had reported that schizophrenia patients present diminished amplitudes and delayed latencies of rod and cone photoreceptor responses (Hébert, Schizophr Res, 2015) and we recently found that bipolar patients have similar ERG anomalies. We had also reported preliminary data in a small sample of 29 children born to an affected parent showing that young offspring had rod diminished amplitudes (Hébert, Biol Psychiatry 2010).

The present objectives were i) under the hypothesis that offspring would display many of the ERG anomalies that schizophrenia or mood disorder patients carry (Hébert, Schizophr Res 2015; Prog Neuropsychopharmacol Biol Psychiatry 2017), to look for cone and rod response anomalies in a large sample of young high-risk offspring; ii) to describe the relationship between ERG anomalies and other risk endophenotypes in the offspring; and iii) look at the relationship between ERG anomalies and the risk clusters already shown to predict later transition to illness.

**Methods:**

The sample consisted of 84 young offspring (aged 6 to 27) of a parent affected by schizophrenia or bipolar disorder, compared to 224 healthy controls balanced for age and sex. Full-field cone and rod ERG was measured in non-dilated eyes for all subjects. In the young offspring, we also collected measures of different cognitive domains, attenuated symptoms of psychosis, non-psychotic DSM diagnosis and/or an episode of poor GAF functioning in childhood-adolescence, childhood trauma, and cannabis use (Paccalet, Schizophr Res 2016).

**Results:**

In comparison to controls the offspring displayed three ERG anomalies that were observed in adult patients: prolonged cone b-wave latency (p=0.04), diminished rod b-wave amplitude (p=0.04) and prolonged rod b-wave latency (p=0.006). These ERG anomalies were shared by offspring of a parent with schizophrenia or bipolar disorder, an observation of ERG commonality that we had made in adult patients. The three ERG amplitude and latency anomalies tended to aggregate in a child at risk, a trend we also observed in another endophenotype modality such as deficits in different cognitive domains. However, in these high-risk children and adolescents, the patterns of aggregation suggest that ERG anomalies would depict another risk pathway than that marked by cognitive deficits.

**Discussion:**

First, ERG anomalies in high-risk children have neurobiological implications for future research on the illness neurodevelopment. Second, as found for other modalities of risk endophenotypes in children at genetic risk (Maziade, New Eng J Med 2017), multiple rod and cone ERG anomalies tended to cluster together in a child. Such an aggregation may be compatible with the multifactorial polygenic theory with a threshold. Remarkably, a clustering of risk indicators is also observed in children at risk of metabolic cardiovascular disorders and is presently considered in practice guidelines for these children. The clustering of risk indicators may provide an empirical basis for the staging of the risk status of children at genetic risk and has immediate implications for their longitudinal surveillance in the clinic.

